# ADP-ribosylation of DNA and RNA^☆^

**DOI:** 10.1016/j.dnarep.2021.103144

**Published:** 2021-09

**Authors:** Joséphine Groslambert, Evgeniia Prokhorova, Ivan Ahel

**Affiliations:** Sir William Dunn School of Pathology, University of Oxford, Oxford OX1 3RE, United Kingdom

**Keywords:** ADP-ribosylation, DNA modification, RNA modification, PARP, DNA damage response

## Abstract

ADP-ribosylation is a chemical modification of macromolecules found across all domains of life and known to regulate a variety of cellular processes. Notably, it has a well-established role in the DNA damage response. While it was historically known as a post-translational modification of proteins, recent studies have shown that nucleic acids can also serve as substrates of reversible ADP-ribosylation. More precisely, ADP-ribosylation of DNA bases, phosphorylated DNA ends and phosphorylated RNA ends have been reported. We will discuss these three types of modification in details. In a variety of bacterial species, including *Mycobacterium tuberculosis*, ADP-ribosylation of thymidine has emerged as the mode of action of a toxin-antitoxin system named DarTG, with the resultant products perceived as DNA damage by the cell. On the other hand, mammalian DNA damage sensors PARP1, PARP2 and PARP3 were shown to ADP-ribosylate phosphorylated ends of double-stranded DNA *in vitro*. Additionally, TRPT1 and several PARP enzymes, including PARP10, can add ADP-ribose to the 5’-phosphorylated end of single-stranded RNA *in vitro*, representing a novel RNA capping mechanism. Together, these discoveries have led to the emergence of a new and exciting research area, namely DNA and RNA ADP-ribosylation, that is likely to have far-reaching implications for the fields of DNA repair, replication and epigenetics.

## 1 Introduction – ADP-ribosylation and the DNA damage response

The ability to efficiently detect and repair DNA lesions is crucial for the maintenance of genomic integrity. Genomic stability is constantly challenged by exogenous and endogenous threats. Indeed, it has been estimated that a cell could experience up to 10^5^ lesions in a day [1]. Cells have thus evolved numerous signalling pathways in order to identify, signal and repair these lesions, collectively referred to as the DNA damage response (DDR). ADP-ribosylation, a chemical modification of macromolecules found across all domains of life, has emerged as a crucial regulatory process of the DDR [2,3].

Chemically, ADP-ribosylation consists in the enzymatic transfer of an ADP-ribose moiety from NAD^+^ onto target substrates with the release of nicotinamide [4]. The modification has been best characterised as a post-translational modification (PTM) of proteins that regulates a variety of cellular processes in addition to DNA repair, including chromatin remodelling, transcription, cell differentiation, anti-viral response, RNA metabolism and cell death [5,6]. ADP-ribosylation is catalysed by the ADP-ribosyltransferase (ART) superfamily of enzymes [7]. ARTs are classified in three families, the diphtheria toxin-like ARTs (ARTDs) also referred to as the poly(ADP-ribose)polymerases (PARPs) [8], the cholera-toxin like ARTs (ARTCs) and sirtuins [9]. PARPs, the most intensively studied ART family, is composed of 17 members in humans, named from PARP1 to PARP16 (two tankyrase enzymes are sometimes referred to as PARP5a and PARP5b) [10]. PARPs can be characterised as either catalysing mono(ADP-ribosyl)ation (MARylation) or poly(ADP-ribosyl)ation (PARylation). In the latter reaction, the amino-acid linked ADP-ribose moiety is extended to form long, often branched, chains [5,11]. Only PARP1, PARP2 and PARP5a/b (tankyrase1/2) have been shown to catalyse PARylation [4].

The best established cellular function of PARPs is its role in the DDR. PARP1, the main ADPr “writer”, PARP2 and PARP3, are swiftly recruited to sites of DNA damage and are thus described as DNA damage sensors [12]. Binding of PARP1−3 to single- and double-stranded DNA breaks (SSBs and DSBs, respectively) leads to a conformational change which induces the relief of the autoinhibitory state [[13], [14], [15], [16], [17]]. Once activated, PARP1−3 will attach poly-ADP-ribose (PAR) chains on many protein targets including themselves, histones, DNA repair proteins and chromatin remodelling factors [18,19]. This DNA-damage induced PARylation triggers a variety of downstream events, including recruitment and assembly of DNA repair machineries as well as chromatin decondensation that promotes the access of repair proteins to DNA damage sites [18,20,21].

Historically, PARP-catalysed ADP-ribosylation was thought to be attached mainly to glutamate and aspartate residues [[22], [23], [24], [25]]. However, further mass spectrometry studies led to a breakthrough in the field and established serine as the major ADP-ribose acceptor residue under both physiological and DNA damage conditions [[26], [27], [28], [29]]. This modification is now well-understood mechanistically and is performed by PARP1 or PARP2 forming a joint active site with Histone PARylation Factor 1 (HPF1), an accessory factor that switches PARP1 and PARP2 substrate specificity towards serine residues [[30], [31], [32]]. Furthermore, the PARP2-HPF1 complex was shown to bridge two DSBs in a conformation compatible with DNA ligation, uncovering the first step of DSB repair [33]. In this bridging conformation, the PARP2-HPF1 complex was still in a competent state for ADP-ribosylation of neighbouring histones and DNA repair proteins [33]. Of note, because of their critical role in the DDR, PARP1 and PARP2 have emerged as important anticancer drug targets, with several PARP inhibitors now used against breast, ovarian, pancreatic and prostate cancers in the clinic [34,35].

Timely removal of PAR chains is crucial to prevent trapping of proteins recruited to the sites of DNA damage and allow access for the downstream repair factors [12]. The reversal of ADP-ribosylation is thus a tightly regulated process catalysed by enzymes belonging to two distinct families, the macrodomains and the (ADP-ribosyl)hydrolases (ARHs) [36]. PARG, a macrodomain enzyme, is the major cellular PAR hydrolase cleaving the ribose-ribose bond linking PAR subunits [37,38]. However, PARG is unable to cleave the bond that attaches the first mono-ADP-ribose (MAR) unit to the target protein [39]. ARH3 is the hydrolase that specifically removes serine-linked MAR synthesized by PARP1/2 in complex with HPF1 [40]. Together, HPF1 and ARH3 modulate serine-linked ADP-ribosylation of several hundred factors involved in the DDR, including DNA-PKcs, XRCC1, FANCI, BRCA1, Polβ, DNA ligases and high-mobility group proteins, and thus have emerged as key regulators of the DDR [26,30,41]. The other hydrolases that have been characterised include macrodomain-type TARG1, MacroD1 and MacroD2 [42]. They were shown to reverse MARylation linked to glutamate and aspartate residues *in vitro*, but their physiological function remains to be elucidated [43,44].

**Fig. 1:**
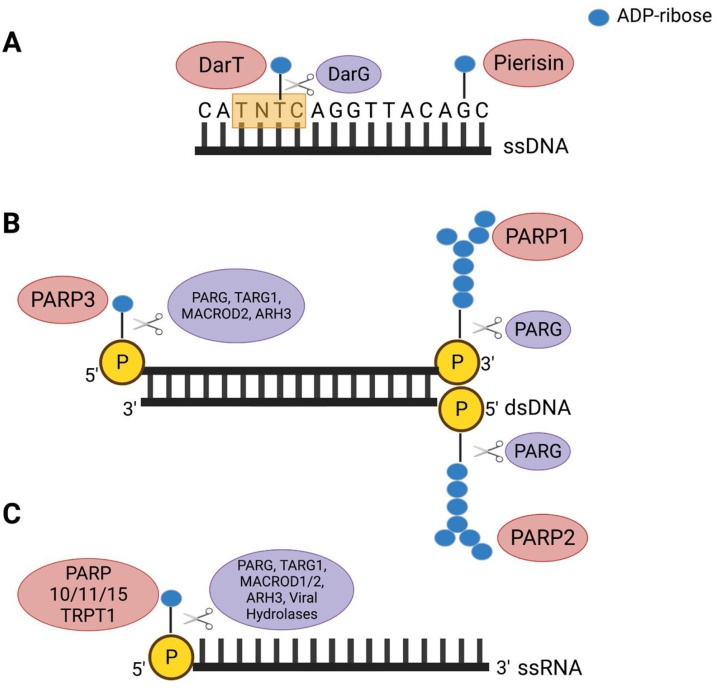
ADP-ribosylation of nucleic acids. (A) ADP-ribosylation of DNA bases. DarT MARylates the second thymine base of a TNTC motif on ssDNA and this modification can be removed by DarG. Pierisin from the cabbage butterfly and its orthologue MARylate guanine on ssDNA in an irreversible manner. (B) ADP-ribosylation of DNA ends. PARP1 and PARP2 PARylate phosphorylated termini on dsDNA with a preference for the 3’- and 5’-terminal phosphate, respectively. The PARP1/PARP2-mediated DNA modification can be removed by PARG. PARP3 MARylates phosphorylated termini on dsDNA with a preference for the 5’-terminal phosphate. This modification can be reversed by PARG, TARG1, MacroD2 and ARH3. (C) ADP-ribosylation of RNA ends. PARP10/PARP11/PARP15 and TRPT1 MARylate ssRNA at the 5’-terminus, forming a non-canonical cap. PARP10 was also shown to modify the 3’-terminal phosphate of ssRNA, albeit less efficiently than at the 5’-terminus. The modification catalysed by PARP10 and TRPT1 was shown to be reversed by PARG, TARG1, MacroD1, MacroD2 and ARH3. Viral macrodomain-containing hydrolases could reverse the modification catalysed by PARP10 on ssRNA 5’-phosphorylated ends.

Despite its crucial role as a PTM in the DDR, ADP-ribosylation can no longer be considered solely as a protein modification. Over the past five years, ground-breaking *in vitro* studies have established nucleic acids as novel substrates of reversible ADP-ribosylation in bacteria, yeasts, mammals and plants. With these discoveries, a new exciting field has emerged with potential far-reaching implications for the physiological function of ADP-ribosylation. The modification targeting nucleic acids can be divided into three categories: ADP-ribosylation of DNA bases, ADP-ribosylation of phosphorylated DNA ends and ADP-ribosylation of phosphorylated RNA ends (Fig. 1). This review will aim to describe these three types of modification in detail, highlighting their significance in DNA repair and discussing the current models of their cellular function.

## 2 ADP-ribosylation of DNA bases

### 2.1 Pierisin and orthologous toxins

DNA ADP-ribosylation was first reported in 2001 in a study showing that pierisin-1, an ARTC enzyme from the cabbage butterfly, is able to MARylate double-stranded DNA (dsDNA) at the N^2^ position of guanine (Fig. 1) [45]. This cytotoxic modification was proposed to have an antiparasitic function [46]. Subsequently, pierisin-2, -3 and -4, and pierisin orthologues from bacteria and shellfish were found to catalyse the same modification *in vitro* [[47], [48], [49]]. There are no pierisin orthologues in human cells but exogenous expression of pierisins in human cancer cell lines strongly induced apoptosis, further highlighting the toxicity of pierisin-mediated DNA adducts and suggesting its use as a potential anti-cancer therapy [50,51]. No enzymes have yet been found to catalyse the removal of pierisin-mediated ADP-ribosylation of DNA bases, suggesting that the modification could be irreversible. Modifications of macromolecules involved in regulating cellular processes, such as PTMs, are most often removable, thereby allowing a tight control over the downstream physiological effects. In the case of pierisin-mediated ADP-ribosylation, the absence of a cellular reversal mechanism strongly points to a genotoxic defence function of the enzyme, as opposed to a signal transduction role.

### 2.2 DarG/DarT, the first well-characterised system for reversible ADP-ribosylation of nucleic acids

**Fig. 2:**
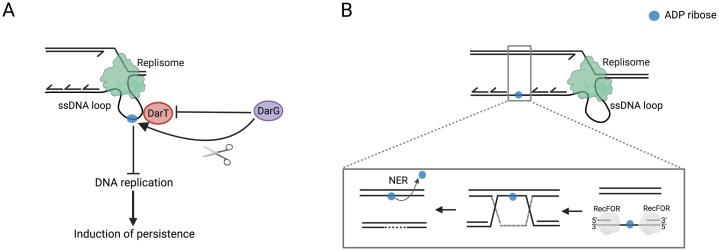
Repair of DarT-catalysed DNA ADP-ribosylation. (A) Direct reversal of DarT-induced DNA lesion by DarG. DarG also inhibits the catalytic activity of DarT through binding and physically sequestering the enzyme. The DarT-mediated DNA lesion inhibits replication which could induce persistence. (B) Model for the DarG-independent repair of DarT-induced DNA lesion. DarT ADP-ribosylates ssDNA loops arising during replication, leading to the generation of single-stranded gaps (SSG). The RecF-mediated homologous recombination repair pathway recognizes the SSG and repairs the gap. The ADP-ribosylated DNA adduct (blue dot) is then repaired by the nucleotide excision repair (NER) pathway.

In 2016, a search for novel ADP-ribosylation enzymes in bacteria led to the discovery of the first well-characterised reversible system for DNA ADP-ribosylation [52]. Several bacterial species, including the human pathogen *Mycobacterium tuberculosis* and enteropathogenic *Escherichia coli* (EPEC), were found to express an operon encoding a toxin-antitoxin (TA) system consisting of a macrodomain and another undefined protein. Structural analysis suggested that this unknown protein could be a highly-divergent PARP-like enzyme [52]. Biochemical characterisation of this protein from extremophile *Thermus aquaticus* led to the discovery of its activity as an ART targeting DNA bases. Specifically, the protein was shown to transfer a single ADP-ribose unit on the second thymidine of a TNTC motif on ssDNA and presents no activity towards dsDNA, RNA or protein substrates (Fig. 1) [52]. Upon discovery of its enzymatic activity, the protein was named DNA ADP-ribosyl transferase (DarT). DarT expression induced growth arrest in EPEC, a phenotype that could be rescued upon co-expression of its partner macrodomain protein, confirming the TA nature of the system [52]. The striking discovery that thymidine ADP-ribosylation catalysed by DarT can be specifically removed by the macrodomain-containing antitoxin, named DNA ADP-ribosyl glycohydrolase (DarG), showed that the pair of proteins exerted its TA function *via* reversible DNA ADP-ribosylation (Fig. 1) [52]. Moreover, DarG is one of the three antitoxins essential for the survival of *M. tuberculosis* [53]. This essentiality was shown to be dependent on DarT presence, further establishing the two proteins as the DarTG TA system [54]. In addition to its ability to remove DarT-mediated DNA ADP-ribosylation, DarG also counteracts DarT activity by physically sequestering the toxin through binding with its C-terminal domain (Fig. 2) [55]. Of note, DarG displays surprising structural homology to the eukaryotic ADP-ribosyl glycohydrolase TARG1 [43,52]. Mutating DarG K80, the residue that corresponds to TARG1 main catalytic residue, completely abolished DarG enzymatic activity highlighting a conserved catalytic mechanism between the two enzymes [43,52].

### 2.3 The DarT-mediated modification is perceived as DNA damage by the cell

The highly toxic effects of DarT expression rescued upon co-expression with DarG strongly suggested that ADP-ribosylation of thymidine was a novel DNA lesion. DarG, specifically removing the thymidine-linked lesions, can thus be described as a non-canonical DNA repair enzyme. An analogy can be drawn with another type of DNA adducts – DNA adenylation that occurs on 5’-phosphorylated DNA upon abortive ligation and is reversed by a specialised DNA repair factor, aprataxin, that is conserved from yeasts to humans [56,57]. Furthermore, the tight control of this site-specific DNA ADP-ribosylation lesion by the DarTG pair could predictably be exploited for targeted induction of DNA damage to control cell metabolism. The DNA damage/repair nature of the DarTG TA pair is supported by the lethal effects of DarG depletion in *M. tuberculosis* indicating that lesion accumulation, in the absence of the corresponding repair enzyme, can induce cell death [54]. Notably, this also suggests that DarG could be a promising drug target for diseases caused by pathogens expressing this TA system, especially tuberculosis. Furthermore, DarG partial depletion was shown to sensitize cells to DNA-damaging agents, suggesting that similarly to guanine-linked ADP-ribosylation described above, thymidine-linked ADP-ribosylation disrupts DNA metabolism pathways [54].

The growth arrest phenotype observed upon DarT expression could be explained by its effect on DNA replication. DarT could potentially target, as its substrate, ssDNA loops arising during replication on the lagging strand, which would impede DNA replication (Fig. 2) [58]. Indeed, expression of DarT led to replication fork stalling in *Thermus aquaticus* and EPEC, supporting the hypothesis that ssDNA loops generated during replication can be the targets of DarT ADP-ribosylation activity [52,55].

Further characterisation of the cellular response to DarT-mediated DNA ADP-ribosylation confirmed that the modification was perceived as DNA damage and that certain cellular repair pathways could reverse the adducts attached to thymidine independently of DarG [55]. In EPEC, the modification catalysed by DarT, which shows a slightly different specificity, preferentially targeting the sequences TTT or TCT on ssDNA, was shown to activate the SOS response [55]. Furthermore, strains unable to initiate the SOS response presented increased sensitivity to DarT expression, indicating that this bacterial repair pathway also contributes to survival upon exposure to thymidine-linked ADP-ribosylation [55]. Moreover, DarG was shown to interact with DNA repair factors such as RecA, RecB and RecF, suggesting that these proteins could be recruited to the sites of DNA ADP-ribosylation along with DarG to mediate repair of these novel lesions [54].

Genetic experiments revealed that a set of DNA repair genes was important for bacterial survival upon DarT expression, pointing to the existence of an endogenous pathway that could recognize and repair this novel DarT-mediated DNA modification in the absence of DarG [55]. Specifically, deletion of genes in the RecF-mediated homologous repair (HR) pathway, involved in repairing ssDNA gaps, significantly reduced bacterial survival [55,59]. RecF-mediated HR would not remove the DNA adduct but rather would transfer the lesion from ssDNA to dsDNA upon strand invasion and subsequent Holliday junction resolution [59]. It was shown that deletions of genes in the nucleotide excision repair (NER) pathway, a pathway activated by the SOS response and involved in removing adducts causing DNA distortions, also induced a significant decrease in cell survival upon DarT expression [55,60]. These results led to establishing the following model for metabolism of these novel DNA lesions: during replication, DarT targets ssDNA loops and the resulting DNA adducts are then recognized by RecF which converts these ssDNA lesions into DNA duplexes; these duplexes are then repaired by the NER pathway (Fig. 2) [55]. As the RecF-mediated HR and NER pathways are conserved in humans, it would be interesting to explore their role in recognizing and removing putative endogenous DNA ADP-ribosylation in human cells.

### 2.4 Models for the cellular function of DarTG

While the studies discussed here have clearly established the toxic effect of DarT-mediated DNA ADP-ribosylation, the tight regulation of the modification by DarG makes it highly unlikely that it is solely a detrimental lesion that does not carry a physiological function. Additionally, the essentiality of the DarTG system for *M. tuberculosis* viability described above and our unpublished observations suggesting that the DarTG TA pair is conserved in all strains of *M. tuberculosis* clearly indicate a key function of the DarTG TA system in the life cycle of this pathogen. Based on its effects on growth and viability, it has been proposed that DarT activity could trigger persistence (Fig. 2), a dormancy-like state that has been involved in inducing antibiotic resistance in the clinic [52,61]. Antibiotic and environmental pressures could trigger DarT activation and induce this dormant state. Upon relief of these pressures, the enzymatic activity of DarG would promote return to normal activity. Furthermore, DarG depletion resulted in increased mutability which supports the hypothesis that the DarTG TA system contributes to the adaptation to changing environments and promotes antibiotic resistance [54]. Thus, in having a putative role in the induction of persistence, DarT emerges as a promising drug target to combat antibiotic resistance. It can be hypothesized that the initiation of the persistent state relies on the loss of DarG activity. In this scenario, the discussed RecF-mediated HR repair pathway coupled with NER would enable the cell to tolerate this toxic DNA lesion in the absence of DarG (Fig. 2). Lastly, a potential role for DarTG in antiphage defence cannot be excluded. DarT specificity for ssDNA could enable the enzyme to differentiate between host and invading viral DNA. Interestingly, DarTG is often encoded together with restriction-modification systems [52]. This could indicate the existence of a novel defence pathway whereby DNA ADP-ribosylation and DNA methylation function together to provide immunity against phages.

While DNA ADP-ribosylation catalysed by DarT remains to be detected *in vivo*, there is accumulating evidence that DarT targets cellular DNA and the resultant products of the reaction are perceived as DNA damage by the cell. At this stage, the DarTG pair is the best characterised system for reversible DNA ADP-ribosylation and its discovery shed light on new aspects of the cellular function of ADP-ribosylation.

## 3 ADP-ribosylation of DNA ends

### 3.1 PARP and PARP-like enzymes can catalyse the reversible ADP-ribosylation of phosphorylated DNA ends

Recent work has extended DNA ADP-ribosylation activity to the well-characterized PARP family. PARP1 and PARP2 were the first mammalian enzymes to be shown to PARylate DNA substrates *in vitro*, creating PAR-DNA adducts (Fig. 1) [62]. Shortly after, PARP3 was found to catalyse MARylation of DNA oligonucleotides (Fig. 1) [43]. The three enzymes were shown to add the modification to phosphorylated DNA ends [62,63]. PARP1 preferentially modifies the 3’-terminal phosphate of a DSB on gapped DNA duplexes [64]. On the other hand, PARP2 preferentially adds PAR chains on the 5’- terminal phosphate of a dsDNA containing a 5’-phosphorylated nick, a substrate preference shared by PARP3 [63,65]. Moreover, when incubated with substrates harbouring two nicks on the same strand, PARP2 was shown to catalyse ADP-ribosylation on the 5’-phophorylated nick in addition to the 5’-phosphorylated DSB termini [65]. The main difference between PARP2 and PARP3 substrate specificities is that PARP3 is unable to target the 5’-phosphorylated DSB of recessed DNA duplex with a double-stranded part [62,65]. Additionally, human tRNA 2'-phosphotransferase 1 (TRPT1), a member of the TPT1/KtpA family and a highly diverged bacterial PARP homologue, sometimes considered the 18^th^ member of the PARP family, was shown to ADP-ribosylate 5’- phosphorylated ssDNA ends [66,67]. TRPT1 homologues in fungi, archaea and bacteria were also shown to possess DNA ADP-ribosylation activity *in vitro*, establishing DNA ADP-ribosylation as a conserved activity of TRPT1 [66,68].

ADP-ribosylation of phosphorylated DNA ends by PARPs is a reversible process. *In vitro*, the DNA modification catalysed by PARP1 and PARP2 was shown to be removed by PARG [62], while PARP3-catalysed MARylation was reversed by PARG, TARG1, MacroD2 and ARH3 [63]. Lastly, TRPT1-mediated DNA modification can be hydrolysed by PARG, TARG1, MacroD1, MacroD2, ARH3 and NUDT16, a phosphodiesterase enzyme [66,69].

This reversible PARP-mediated ADP-ribosylation was also observed for plant enzymes. A recent study showed that PARP1 and PARP2 from the flowering plant *Arabidopsis thaliania* (atPARP1 and atPARP2) possessed *in vitro* ADP-ribosylating activity towards 5’-phosphorylated ends of DNA [70]. AtPARP1 preferentially targets the terminal 5’-phosphate on recessed DNA duplexes, whereas atPARP2 preferentially modifies 5’-phosphate of nicked and gapped dsDNA substrates. The DNA modification is also reversible as *A. thaliania* PARG can catalyse its removal. These finding suggest that reversible ADP-ribosylation of DNA is an evolutionary conserved activity of PARPs across eukaryotic species, but the physiological relevance of these modifications remains unknown.

### 3.2 *In vitro* characterisation of the DNA ADP-ribosylating activity of PARPs

Further *in vitro* characterisation of the DNA ADP-ribosylation activity of PARP1−3 showed that these reactions require its oligonucleotide substrate to harbour at least two SSBs and the distance between the breaks was shown to strongly influence the reaction efficiency [64,65]. Mechanistically, this can be explained by the role of DNA breaks as allosteric activators of PARP1−3, whereby binding of DNA breaks to the DNA-interacting domain of these enzymes induces the relief of the autoinhibited state to promote ADP-ribosylation [[13], [14], [15]]. Based on structural studies, the distance between the active site and the DNA interacting domain is likely to be too big for a PARP activating DNA break to also act as a target for ADP-ribosylation [16,71]. However, a second break on the same molecule situated at the appropriate distance can serve as a substrate for the PARP-catalysed reaction. Another factor that might influence PARP enzyme activities is their dimerization status. *In vitro* experiments on PARP2 have shown that the monomeric enzyme preferentially binds to nicked DNA, while dimers favour blunt-end DNA [71]. How these two binding modes affect DNA ADP-ribosylation remains to be explored.

The fact that DNA acts both as an allosteric activator and a substrate creates a selectivity filter whereby only DNA molecules with the optimal distance between the two breaks can be modified. *In vitro* studies of PARP1−3 have shown this distance to be between one and two helix turns [64,65,72]. At this stage, the best *in vitro* substrate identified for PARP1-mediated DNA ADP-ribosylation is a 3’-terminal phosphate on a DNA hairpin with a 1 nucleotide-gap on the opposite strand separated by a distance of 13 nucleotides [64]. Strikingly, when PARP1 is incubated with this optimal substrate, ADP-ribosylation of phosphorylated DNA ends is more efficient than automodification, which is considered to be the principal activity of this enzyme [64]. Similar results were obtained upon PARP2 and PARP3 incubation with their respective optimal *in vitro* DNA ADP-ribosylation substrate [65]. In addition to suggesting that DNA ADP-ribosylation could be abundant *in vivo*, these results may also be physiologically relevant since PARP1−3, as DNA damage sensors, interact with both DNA and proteins in the cell. It can be hypothesized that the substrate preference of PARP1−3 for DNA is regulated by an unidentified co-factor that would mediate efficient and specific modification of phosphorylated DNA termini by the enzymes. Furthermore, as it was recently shown that bridging of two DNA breaks by PARP2-HPF1 activates PARP2 PARylation activity on proteins, it would be interesting to establish whether such activating mechanism also exists for the PARylation of DNA ends [33].

### 3.3 Models for the cellular function of ADP-ribosylation of DNA ends

While the above studies mainly focused on characterising novel DNA ADP-ribosylation enzymatic activity *in vitro*, the concentration of NAD^+^ used in the assays suggests that these reactions could also exist in cells. Indeed, the PARP1 ADP-ribosylating activity of phosphorylated DNA ends was observed in a 2–1000 μM NAD^+^ concentration range, encompassing the nuclear and cytoplasmic NAD^+^ concentration, estimated to be around 100 μM [64,73]. Moreover, the DNA substrates with phosphorylated termini used in these studies could be found in cells, resulting from the direct effects of reactive oxygen species on DNA [74]. Additionally, they could also be intermediates of DNA repair or DNA replication. For instance, DNA duplexes with a SSB in proximity to a DSB could arise during the HR and non-homologous end-joining repair pathways [75], while recessed DNA duplexes can be generated by replication fork collapse [76]. Moreover, the substrates in these studies harbouring phosphorylated nicks and gaps mimic base excision repair intermediates or could arise as a result of abortive topoisomerase activity [77,78]. These considerations further support the possibility of these reactions occurring *in vivo*. Several experiments were performed to explore this hypothesis. Upon incubation with HeLa or HEK293 cell nuclear extracts, DNA oligonucleotides harbouring phosphorylated DNA ends were shown to be ADP-ribosylated [64,65,72]. Nuclear extract experiments also suggested that PARP1 contributes to the majority of detected ADP-ribosylation of phosphorylated DNA ends in the cell [65]. This is consistent with PARP1 being the most abundant PARP in mammalian cells and catalysing the majority of PARylation upon DNA damage [79]. Furthermore, preliminary *in vivo* assays suggested that this DNA-linked modification could be indeed detected on genomic DNA [65,70]. Altogether, these results strongly point to the existence of DNA ADP-ribosylation reactions *in vivo*. The unbiased detection of endogenous ADP-ribosylated DNA remains technically challenging. Developing methods that can specifically detect this modification could lead to significant breakthrough in establishing DNA ADP-ribosylation as a physiological process.

It has been suggested that ADP-ribosylation of terminal phosphates could be a DNA lesion resulting from erroneous PARP activity [63]. In this case, ADP-ribosyl glycohydrolases such as PARG and TARG1 could be seen as direct DNA repair factors that remove such potentially cytotoxic DNA adducts [63]. Similarly to DarG in bacteria, the analogy can be drawn with DNA adenylation that occurs upon abortive ligation and is reversed by aprataxin [56,57].

On the other hand, DNA ADP-ribosylation could itself be involved in the DDR, similarly to protein ADP-ribosylation, facilitating the recruitment of DNA repair factors and promoting DNA repair. Additionally, DNA ADP-ribosylation could interfere with the activity of DNA processing enzymes such as DNA helicase, pausing replication and thus making time for efficient DNA repair. DNA ADP-ribosylation could also promote error-free repair by interfering with the binding of factors involved in error-prone DNA repair pathways, *e.g.* the Ku70/Ku80 heterodimer that binds to DNA ends and initiates the non-homologous end joining repair [80]. It has also been suggested by *in vitro* assays that MARylation catalysed by PARP3 on the 5’-phosphate group of gapped DNA could serve as a substrate for DNA ligases [72]. In the cell, this modification could facilitate DNA ligation and thus accelerate DNA damage repair, promoting genome integrity. Alternatively, in being attached to the terminal phosphate, ADP-ribosylation could protect DNA ends from unregulated nuclease activity. Indeed, *in vitro* experiments showed that the oligonucleotide substrates were protected from calf intestinal phosphatase (CIP) activity upon modification by PARP1−3, suggesting that the modified end might also be inaccessible to exonucleases [62,63]. Nevertheless, at this stage, all these attempts to describe the physiological function of ADP-ribosylation on phosphorylated DNA ends remain speculative as all the mentioned hypotheses await experimental confirmation *in vivo*.

## 4 ADP-ribosylation of RNA ends

### 4.1 PARP and PARP-like enzymes can also catalyse reversible ADP-ribosylation of phosphorylated RNA ends

DNA is not the only nucleic acid substrate that can be ADP-ribosylated. Phosphorylated RNA ends are chemically similar to phosphorylated DNA ends and can also be targeted by this modification *in vitro*, extending the repertoire of substrates for ADP-ribosylation [66,68]. Specifically, certain mammalian PARPs were shown to catalyse this reaction. PARP10 catalytic domain can modify phosphorylated ssRNA ends with a preference for 5’-terminal phosphate (Fig. 1) [66]. Full-length PARP10 can also catalyse the modification albeit less efficiently than the catalytic domain alone, suggesting the existence of an autoinhibited state similar to that of PARP1 [13,66]. Additionally, PARP11 and PARP15 could also ADP-ribosylate 5’-phophorylated ssRNA (Fig. 1) [66]. Homologues of human TRPT1 from fungi, archaea and bacteria can also perform the modification of 5’-phosphorylated ends of RNA (Fig. 1), forming a non-canonical 5’-cap structure as discovered by the Shuman group [68]. Later, the same activity was demonstrated for the human TRPT1 orthologue among others, establishing RNA ADP-ribosylation as a conserved activity of TRPT1 across all domains of life akin to its DNA ADP-ribosylation activity [66]. TRPT1 is known for its essential function in the fungal tRNA splicing pathway in which the enzyme catalyses the transfer of an RNA 2’-monophosphate to NAD^+^, yielding a 2’−OH RNA [81,82]. However, many species expressing a TRPT1 homologue do not possess intron-containing tRNAs or mechanisms that would generate 2’-phosphate RNA, suggesting that instead RNA capping could be a primary activity of many TRPT1 homologues [66].

Similarly to ADP-ribosylation of DNA ends, this novel RNA modification is reversible. PARG, TARG1, MACROD1, MACROD2 and ARH3 are able to remove ADP-ribosylation catalysed by PARP10 on either the 3’- or 5’-phosphate termini of RNA oligos (Fig. 1) [66]. Additionally, TRPT1-mediated 5’-phosphate RNA modification can be reversed by PARG, TARG1, MACROD1, MACROD2, ARH3 and NUDT16 (Fig. 1) [66]. ADP-ribosylation catalysed by the *Streptomyces coelicolor* TRPT1 homologue could also be removed by the *S. coelicolor* MacroD-like ADP-ribosylhydrolase SCO6450. Of note, PARP10 expression is induced by interferons and was shown to inhibit the replication of the Venezuelan equine encephalitis virus (VEEV) and other alphaviruses [83,84]. This prompted Munnur et al. to test the hydrolytic activity of macrodomain-containing ADP-ribosylhydrolase from VEEV, as well as severe acute respiratory syndrome coronavirus (SARS-CoV), towards ADP-ribosylated RNA. Strikingly, both the VEEV and SARS-CoV hydrolases could reverse the PARP10-mediated RNA modifications (Fig. 1) [66]. Notably, this activity is also conserved in the homologous hydrolase SARS-CoV-2 [85].

### 4.2 Models for the cellular function of ADP-ribosylation of RNA ends

RNA modification by TRPT1 and PARP10 rendered the oligonucleotide substrates resistant to CIP treatment, indicative of an RNA capping mechanism [66,68]. This non-canonical RNA cap could potentially enhance RNA stability by protecting its ends from nuclease degradation. It could also recruit proteins involved in RNA signal transduction similarly to m^7^GpppN mRNA cap recruiting eIF4E to induce translation initiation [86]. An interesting parallel can also be drawn with another type of non-canonical capping found in mammals, bacteria and yeasts whereby the whole NAD^+^ moiety is attached to the RNA 5’-end, which has been shown to promote RNA decay in mammals [[87], [88], [89], [90]].

Multiple results suggest that PARP10-mediated modification of RNA ends could have an immune function. Firstly, PARP10 expression was shown to inhibit VEEV translation and replication [83,84]. Furthermore, PARP10 was also shown to regulate NF-κB signalling, indicating a potential role in modulating inflammation [91]. Consistent with the antiviral role of ADP-ribosylation, the conserved viral macrodomain encoded within the non-structural protein 3 of alphaviruses and coronaviruses was shown to suppress the innate immune response and promote virulence [92,93]. However, the physiological targets of both PARP10 and the viral hydrolases remain unknown. Based on the exciting findings that both VEEV and SARS-CoV macrodomains can remove ADP-ribosylation from PARP10-modified RNA, it can be hypothesized that PARP10 exerts its immune function by targeting RNA. In encoding ADP-ribosyl glycohydrolases, viruses would have evolved a mechanism to counteract this activity. The viral macrodomains thus emerge as promising antiviral drug targets.

Upon being added on viral RNA, the modification could be recognized by immune factors, having a role in the initiation of the immune response. Additionally, PARP10 inhibitory effects on viral translation could be a direct result of RNA ADP-ribosylation whereby the ADP-ribosylation cap would prevent translation. This hypothesis implies the existence of a mechanism preventing PARP10 from targeting cellular RNA. It has been observed that the RNA recognition motif (RRM) domain contributes to PARP10 catalytic activity [94]. This suggests the presence of a physical link between the catalytic and RRM domains that would enable the latter to modulate PARP10 enzymatic activity [94]. Applying this model to PARP10 immune function, it can be hypothesized that the RRM domain has a role in distinguishing foreign from host RNA. The PARP10 RRM domain could specifically bind to invading foreign RNA, triggering PARP10 activation and leading to specific ADP-ribosylation of viral RNA. However, further experimental work is needed to validate these models.

## 5 Concluding remarks

ADP-ribosylation can no longer be solely regarded as a PTM. Several ARTs targeting DNA and RNA in a reversible manner have now been identified including DarT for the MARylation of thymine bases, PARP1−3 for the modification of phosphorylated DNA ends, and PARP10, PARP11 and PARP15 for the MARylation of phosphorylated RNA ends. While these modifications have not yet been detected *in vivo* due to technical challenges, there is strong evidence that ADP-ribosylation is a widespread nucleic acid modification across all domains of life. This suggests that ADP-ribosylation of DNA and RNA could both contribute to the well-characterised physiological effects of the modification, as well as reveal its new cellular functions. Similarly to the recent discoveries of HPF1 and serine ADP-ribosylation [10,26,29,30], uncovering nucleic acids as additional substrates of ADP-ribosylation forces us to reconsider some of the established models in the ADP-ribosylation field. It can be predicted that ADP-ribosylation of nucleic acids will emerge as a key facet of ADP-ribosylation signalling with important implications for the fields of epigenetics and DNA repair.

## Funding source

10.13039/100010269Wellcome Trust (grant numbers 101794 and 210634), 10.13039/501100000268BBSRC (BB/R007195/1), 10.13039/100014008Ovarian Cancer Research Alliance (Collaborative Research Development Grant #813369) and 10.13039/501100000289Cancer Research UK (C35050/A22284).

## Declaration of Competing Interest

The authors report no declarations of interest.
